# Exploring experiences of implementing standardized cancer patient pathways within investigatory units – a qualitative study

**DOI:** 10.1186/s12913-021-06915-1

**Published:** 2021-09-08

**Authors:** Jeanette Winterling, Sara Delilovic, Jessica Dervish, Malin Gunarsson, Mårten Åhström, Henna Hasson

**Affiliations:** 1 Medical Unit Head, Neck, Lung and Skin, Theme Cancer, Karolinska University Hospital, Stockholm, Sweden; 2 Department of Neurobiology, Care Sciences and Society, Division of Nursing Karolinska Institutet, Stockholm, Sweden; 3 Center for Epidemiology and Community Medicine, Region Stockholm, Stockholm, Sweden; 4 PROCOME research group, Medical Management Centre, Karolinska Institutet, Stockholm, Sweden

**Keywords:** Standardized patient pathways, Cancer, Implementation, Qualitative, Endoscopy, Radiology, Pathology

## Abstract

**Background:**

In the implementation of standardized cancer patient pathways (CPPs), the investigatory units, endoscopy, radiology and pathology, are crucial to ensure an eventual cancer diagnosis. However, when evaluating the implementation of CPPs, little attention has been paid to the healthcare professionals working in these units. The aim of this study was to explore experiences of the implementation of CPPs among health professionals in investigatory units.

**Methods:**

This descriptive qualitative study included 55 health professionals working in investigatory units. Participants were interviewed in 2017–2018, and data were analysed using thematic analysis.

**Results:**

The health professionals reported benefits, facilitators and challenges when describing their experiences of implementing CPPs. Benefits included that CPP improved collaboration and increased focus on the patients. Facilitators in the implementation process included pre-existing well-functioning work processes and having supportive functions (e.g. coordinators). Challenges included the lack of staff and clinical equipment, as well as unjustified time-slots and incorrect referrals.

**Conclusions:**

The findings show that most health professionals working in investigatory units’ experience benefits with the implementation of CPP, but the lack of resources was especially hard to overcome.

**Supplementary Information:**

The online version contains supplementary material available at 10.1186/s12913-021-06915-1.

## Background

Today, the incidence of cancer is increasing, and cancer is one of the leading causes of death in many countries [1]. The shortness of the diagnostic interval, i.e. the period from the first presentation of one or several potential cancer symptoms to cancer diagnosis [2], and the time to start of treatment is crucial for better survival. In addition, a long waiting time increases stress for patients [3]. Navigating the patient through the diagnostic interval demands the involvement of many actors in the healthcare system, including different care units and professions. Investigatory units, i.e. endoscopy, radiology and pathology, provide healthcare professionals in primary and secondary care with the results of cancer investigations, which are crucial to ensure an eventual cancer diagnosis [4].

Standardized cancer patient pathways (CPPs), or fast-tracks, were introduced between 2000 and 2015 in several European countries to resolve the problem of unacceptably long waiting times in cancer care [2, 5–7]. Previous research indicated that CCPs decreased the diagnostic interval in the UK [2] and in Spain [6], inspiring Scandinavian countries to implement them too [4, 5, 7]. Although Sweden has a publicly funded health system and one of the highest survival rates in cancer care in the world, there were large differences in waiting time for a cancer diagnosis, both within and between geographical areas and cancer types [3]. In 2015, Sweden implemented CPPs based on Denmark’s integrated cancer care pathways model [8]. In contrast to Denmark, the Swedish model includes both primary and secondary care [4] and comprises three key concepts: first, manuals with recommended lead-times for each cancer pathway from first symptoms until start of treatment [4, 9]; Second, CPP-coordinators responsible for the administrative aspects of the CPP were located at the units involved in the pathways [10]; and the third key component concerned the introduction of so-called ‘unoccupied time slots’ within investigatory units. This means that the investigatory units, such as endoscopy and radiology, must have pre-booked time slots for investigations of cancer patients to ensure that cancer investigations, i.e. procedures or tests, needed to diagnose cancer, are performed within the given lead-time. For pathology, this meant handling tests marked with CPP more quickly and in accordance with the given lead-time. Thus, how well the investigatory units manage to implement the CPPs is a key factor in the success of the whole care chain.

The goal of implementation science is to improve the quality and effectiveness of care [11]. Obtaining an understanding of the implementation of new processes in healthcare, such as CPP, is crucial. In this study the Consolidated Framework for Implementation Research (CFIR) [12] was used for guidance on what is important to address in implementation science. The CFIR is composed of five major domains that interact with each other and influence the success of the implementation of an intervention; the intervention, outer setting, inner setting, process and the individuals involved [13]. The CFIR framework focuses on identifying information about what works well in the new process and what can be improved in a particular context [13, 14]. An important part of evaluating an implementation is to capture the experiences of the individuals involved [12]. While a previous study has investigated healthcare professionals’ experiences of CPPs [15], no studies have focused on investigatory units. An understanding of how investigatory units manage to change their routines and how they experience the implementation of the CPP is essential both for evaluating the success of the implementation and knowing how to support healthcare professionals when barriers to implementation are identified. The aim of this study was to explore experiences of implementing the CPPs among health care professionals working within investigatory units. Thus, this study makes an important contribution to health service research by highlighting the role of investigatory units in the implementation of CPP**s**. When implementing CPPs all actors’ roles are crucial as they are dependent on each other’s services. Identifying what works well and what can be improved in certain contexts of an implementation process [13, 14], such as the investigatory units, is important for understanding the whole chain.

## Method

### Design

A descriptive qualitative design was adopted and the COREQ-checklist for qualitative studies followed.

### Study setting

This study explores experiences of implementing CPPs among healthcare professionals working in investigatory units: endoscopy, radiology and pathology in Region Stockholm. The staff in these units include; endoscopists, pathologists, radiologists, radiographers, radiology nurses, nurses, assistant nurse and administrative personnel. At the time of the study, there were 31 such units in the region, and they are responsible for handling referrals from mainly primary care but also from emergency and other health care units. With around 2.3 million inhabitants, Region Stockholm has the largest number of inhabitants of the 21 regions in Sweden. The region has an overall responsibility for the care of its inhabitants, including both publicly and privately financed health care providers [16].

### Participants and procedure

To obtain staff experiences both of the initial implementation phase and the on-going work with CCP, participants were recruited at two time-points in 2017 and 2018. The purpose of collecting data at two time-points was to capture perceptions of potential changes during the study period, as changes during an implementation process takes time [12]. The study population comprised health professionals working in any of the investigatory units in Region Stockholm and involved either clinically or administratively with CPPs. Recruitment began in 2017 with the Regional Cancer Centre’s steering group identifying key professionals involved in the CPPs. Thereafter, these persons suggested other suitable professionals to the researchers, who were then invited to participate. Thus, snowball recruitment was used to achieve a purposive sample [17] and the process continued until saturation had been achieved, meaning no new themes were identified in the data [18].

In 2018, follow up interviews with participants were performed and additional snowball recruitment was used to recruit new participants. Furthermore, the register for staff working in the Region Stockholm was scanned to identify new health professionals, who were then invited to the study. The researchers contacted all potential participants by phone or email and they sent them a written invitation to participate. The invitation included information about the purpose of the study, ethical considerations and contact details. A few potential participants did not participate due to lack of time or that they had retired.

In total, 17 interviews were conducted in 2017 and 35 interviews in 2018. All interviews were conducted individually and face-to-face, except in one case, where two individuals took part in the same interview. Altogether, 55 health professionals participated, comprising 47 specialist physicians, five coordinators, two contact nurses and one medical secretary and the number of participants by investigatory unit was: 19 from endoscopy, 21 from radiology and 15 from pathology. No demographic data were gathered since the aim was not to assure representativeness and equal distribution for those parameters.

### Data collection

Three of the authors (JD, MG, MÅ) and four other investigators from the Centre for Epidemiology and Social Medicine performed the data collection. They were both female and male with backgrounds from public health, implementation research and anthropology. The use of several researchers in the data collection and analysis allowed for reflexivity and reduced bias [19]. They all had experience of interviewing and no relationship to the participants. To capture the perspective of the individuals involved in the implementation process of CPPs within the investigatory unit**s** [13] an interview guide was used. It was developed from previous research in the field [4, 20]; it contained questions regarding the participants’ reflections on the implementation and the continued work with the CPPs. The interview guide covered themes such as: experienced barriers and facilitators (both in their unit and across healthcare generally), capacity to work with the CPPs and collaboration in health care. As different healthcare professions have divergent functions in relation to the CPPs, the interview guide for coordinators, nurses and administrative staff included some questions about administrative tasks. The interview guide was modified for the 2018 data collection to focus on the continued work with CPPs, instead of the initial implementation phase (Interview guide is seen as supplementary). The interviews lasted, on average, 30–40 min and were conducted in a private room at the participants’ workplace. All interviews were conducted in Swedish, digitally recorded and transcribed verbatim.

### Data analysis

All interviews were pseudonymized, meaning the names of the participants were replaced with a number followed by a letter. The coded data and the key were saved on locked hardware, only accessible to the researchers. A thematic analysis was conducted to identify, analyse and describe patterns in the data [21]. Through systematic data familiarization, data coding and theme development, coherent patterns emerged. The analysis was carried out in three steps. First, the transcripts were carefully read by two of the authors (JD and MG) to obtain a holistic view of the material and to reduce individual bias. Second, the authors selected quotations in the raw material that were relevant to the research aim, which were then placed in a separate document. Afterwards, the quotations were read and discussed between the two authors to identify concepts, similarities and differences in the material. This was an iterative process, meaning it was done several times to ensure that no information-rich data were overlooked. Third, the similarities, differences and concepts were related to each other, forming the basis of the analysis. The final themes and analysis were then discussed and approved by all authors. Using quotations helped to illustrate the findings and demonstrate the logic behind the data interpretation. Quotations were translated from Swedish to English by the authors and crosschecked by a professional language reviewer.

### Ethical considerations

Ethical approval was obtained from the regional ethics board of Region Stockholm (2017/1328-31). Written consent was obtained from all participants after they had received fully information on the study, including that their anonymity would be protected and that their data would be securely processed. They were also informed about their right to withdraw at any time without further explanation. In the presentation of the findings, all quotations are anonymized.

## Results

**Fig. 1:**
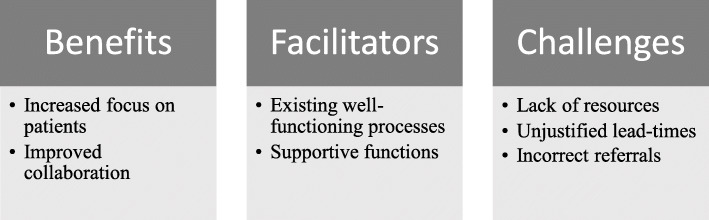
Presentation of the seven themes divided into benefits, facilitators and challenges when implementing and working with CPPs in investigatory units

The analysis resulted in seven themes, some focusing on the benefits of implementing CPP, some describing facilitators in the implementation process, and others identifying challenges in working with CPPs (Fig. 1). Below, each theme is described and illustrated by quotations from different interviews. Quotes are altered to better enhance the respondent’s formulation, without compromising their meaning.

### Increased focus on patients

Participants expressed that, before the CPPs, there was a tendency to regard patients as anonymous samples. Now, there was a far greater focus on patients’ perspectives and experiences of care.

*“You devote more time to thinking about each individual patient. It’s so easy to become anonymous in a laboratory. You only see anonymous samples and not the patient behind them. The patient’s needs, and the complexity of cancer care are raised far more now. You work a little extra and prioritize patients because you see them as people and not as anonymous samples.” (pathology#1)*.

Many described this increased focus on the patient as a long-awaited development in cancer care.

*“It has finally been understood that waiting is torment for patients. A difference compared to before is that now, every patient in a CPP is asked: ‘how did you experience your treatment?’ That’s something that hasn’t been emphasized much earlier, but now it’s very much in focus.” (radiology#11)*.

Moreover, several participants felt that the increased patient focus also had led to a deeper understanding of how the various parts of the care chain were connected and how responsibility for the patients was shared among colleagues in different units. Participants noted how they themselves, as well as colleagues in other units, had become more process-oriented since they started working with CPPs. There was an increased motivation to speed up investigations to reduce long waiting times for patients.

*“Now, the focus is on how you can make investigations faster. If a referral is labelled CPPs, you understand the need to handle it faster. In that way, it has an effect.” (endoscopy#3)*.

### Improved collaboration

Many participants reported that the CPPs contributed to a greater consensus on how the work within cancer care should be carried out, even among the competing private businesses.

*“Obviously, as private X-ray departments, many of us are competitors, but we have opened up to a common vision of what to do and how to do it, which we did not have before.” (radiology#2)*.

It was also emphasized how multidisciplinary conferences, which have become a more integral part of cancer care since the introduction of the CPPs, enabled closer collaboration among different caregivers in the care chain.

*“We work in a fairly integrated way with the units through multidisciplinary decision-making conferences, and as we are also sub-specialized, we have good contact with our referrals.” (pathology#9)*.

Some participants described that they had collaboration with other external units already in place before the CPPs, which they believed constituted a good foundation for the implementation of CPPs.

### Existing well-functioning processes

Many of the participants expressed that they were familiar with working in controlled processes with high demand for quick investigations before implementation of CPPs. They stated that their work structures already functioned well, which facilitated the implementation of CPPs.

*“We have a well-established routine for where and when the referral examination should take place, how it should be done and who should do what. It’s generally a good structure and raises very few questions. We work according to templates, and therefore, we can follow these CPPs very well. That’s our strength.” (endoscopy#8)*.

Some participants from endoscopy stated that there was a noticeable difference when CPPs were implemented compared to their previous way of working, because the CPPs provided them with more structure.

Other participants expressed that nothing had changed since the implementation of CPPs, except that the name used for different things in the work process, i.e. time-slots, was new, but they actually did the same work as before.

*“For those of us who worked in a similar way before CPPs were implemented, there was no major change. We just took the slots we already had, labelled them CPPs and went on with our usual routine.” (radiology#3)*.

### Supportive functions

Many described that CPP coordinators, in particular, and other supportive functions, such as external developers, added during implementation were perceived as fundamental to advancing the work with CPPs. They were described as invaluable to get the CPP in place.

*“These coordinators have an important function. They make the system work more smoothly so that we are able to fix this [the CPPs], as it is a little unpredictable which patient will have cancer and not. It’s a really good function to have.” (endoscopy#4)*.

One of the participants described how using an external developer resulted in a more effective CPP implementation process.

*“One of our chief physicians hired a strategic business developer who did not have any healthcare experience but was really talented when it came to developing effective processes. She standardized the way of working so that everybody in the unit started working in the same way.” (radiology#11)*.

### Lack of resources

In all units, lack of staff and equipment were mentioned as hindrances when working with the CPPs, and these deficits were perceived as stressful and made the work with the CPPs more difficult. At an early stage in the implementation, the investigatory units communicated that it would be impossible to achieve the goal of the CPPs without the necessary resources. However, many felt that this feedback was not considered by those responsible for management of the CCPs.

*“There was already some frustration when the CPPs were introduced. In the initial phase of the CPP [implementation], it was already claimed from our side that this isn’t something we can achieve with existing resources, it is a dream scenario that requires the provision of resources to be in place.” (radiology#3)*.

Furthermore, there was a shortage of several crucial occupational categories, including nurses, doctors and biomedical analysts. The staff shortage became particularly problematic during holidays and absence due to sickness. There was also a lack of clinical supervisors for the junior medical doctors. Not receiving the time needed for supervision made it difficult for new doctors to build on their expertise and handle more advanced patient cases.

*“There is a shortage of radiologists. It’s a problem that takes time to fix, and now we have had to hire more doctors, which in itself is positive. At the same time, they must also have supervision and there is a shortage of supervisors.” (radiology#7)*.

Moreover, the lack of equipment hampered work with the CPPs. Pathologists highlighted the wish that digital diagnostics would soon be implemented at all pathology units in Sweden. This would make it possible to use competence from the few existing pathologists in the CPPs, and they believed that this could lead to shorter lead times and a more equal quality of care across the whole country. However, the introduction of digital pathology seemed to progress slowly in several units.

*“Every analysis needs one or more people, and each analysis costs and takes time. There is a new machine model that can gather all these analyses and leave a response, which can help us save personnel, time and costs.” (pathology#2)*.

Meeting the growing need to achieve the requirement for shorter lead-times was sometimes described as frustrating by the participants. Lead-times were difficult to maintain due to lack of resources, holidays and that preparations sometimes needed to be complemented by additional analyses and second opinions from colleagues. For instance, in most cases, endoscopic examinations required preparation for patients that went beyond the established ten calendar days.

*“This time requirement, that it should be done within ten calendar days, it’s not even ten working days, that’s an extremely tough criterion if one takes into account how we usually examine referrals.” (endoscopy#1)*.

### Unjustified lead-times

There was a concern about whether the CPPs’ strict time requirements were always medically justified, especially in terms of slow-growing tumours. Several participants were critical of the idea that faster investigations yielded better results and argued that useful answers could, in fact, justify longer waiting times. Speeding up investigations that were not medically urgent was difficult to justify, given the widespread staff and resource shortages.

*“As an experienced pathologist, I know that a disease has its natural course, that is, if you get a response a few weeks later it has no effect on the cancer. I prefer to give an accurate answer that ‘yes that was microscopic colitis.’ Then you get the right treatment, you come to the right doctor, you know what to do. If you do a good quality job, it ultimately benefits the patient and that must take time.” (pathology#5)*.

### Incorrect referrals

Finally, participants addressed the difficulties that emerged when CPP referrals were mislabelled or incomplete, mainly from primary care but also from other healthcare providers. For example, patients without malignancy were sometimes labelled CPPs, while patients with malignancy were not, leading to the risk that cancer may not be detected in time. The problem also caused an unnecessary influx of patients, so that those with malignancy had to wait longer for investigation. Therefore, the participants argued that they could not assume that only the marked referrals should be dealt with promptly and they felt the need to be observant.

*“We still think there is a certain gap, or maybe we have interpreted the message differently. How do you label these samples that come primarily from primary care and what is it that you label and how do you keep the CPP separate from acute marking?” (pathology#8)*.

Another common error was that referrals lacked relevant information and contact details were missing. Consequently, questions arose, and it was difficult for the investigatory units to contact patients.

*“Something that’s often missing when we receive the referrals and you have to handle them within five days is contact information for the patient and their telephone number. That should be a mandatory field in a CPP referral, because otherwise it can take a long time to get hold of the patient. It may take several days just to book them for an appointment. We don’t have many extra times that we can set aside. We are often fully booked so it becomes a problem.” (radiology#13)*.

## Discussion

Health professionals expressed that one benefit of implementing CCPs was that it had enhanced focus on the perspective of the patient in their process of receiving or not receiving a cancer diagnosis. It is interesting that this perspective was not described in the earlier Swedish study in which healthcare professionals from all sorts of units involved in the CPP were interviewed, i.e. not only investigatory units [15]. This is probably due to the very limited number and length of face-to-face meetings between investigatory unit staff and patients, which makes investigatory staff more unaware of the patients’ overall experience of the diagnostic interval, compared to other health professionals in the health chain. The increased patient focus, which is one of the core factors of the CPP, strengthened healthcare professionals’ motivation to speed up investigations to reduce unjustly long waiting times. This had been observed in other evaluations of CPP implementation [6, 7]. The motivation of health professionals to reduce waiting times may also be increased when politicians actively start to discuss and set goals for waiting times, which happens in conjunction with CPP implementation [7].

Another benefit of implementing CCP was that health professionals expressed that they had gained a deeper understanding of how the various parts of the care chain were connected and the shared responsibility of colleagues and units for each patient’s pathway. Such increased collaboration between units is also described in other research [4, 9, 15, 22]. It is important to highlight that some participants in the present study expressed that implementing CPPs had not led to any major difference in how they worked because they already had well-functioning work processes, good collaboration and a patient-centred focus. This can be compared with results from the evaluation of the implementation of fast-tracks in cancer care in Spain [6], where health professionals initially expressed scepticism about implementing CPPs because it simply meant formalising something that already existed informally, but later in the implementation process, they changed their view and stated that working harder to deal more promptly with investigations in order to obtain results for patients with suspected cancer had become more important [6]. The CPP coordinators were, in the present study, described to play an important role in achieving the benefits of CPPs. As described in other research, this was due to their responsibility to have close contact with all units involved in the patients’ cancer diagnosis process [23], to provide patients with information about what will happen next and within which timeframe [4], as well as to guarantee continuity of care in each patient’s trajectory [23–25].

The results of the present study also revealed that health professionals in investigatory unit’s experience challenges when working with CPP. The biggest challenge was the lack of resources, which was clearly frustrating and has also been described by health professionals from other units involved in CPP in Sweden [15]. This is not surprising, as CPP was implemented at a time when diagnostic capacity, e.g. mainly radiology and pathology, failed to meet current demand [9, 10, 26] and these units were therefore identified as bottlenecks [26]. Similar problems are reported from the UK, where it was estimated that 10–20 % of investigations or appointments had to be rescheduled because the results from radiology or pathology were not available in time [27]. Although work processes in these units in Sweden were streamlined and new forms of collaboration between different stakeholders were established to enable adherence to the time frames set out in the manuals [4, 9, 26], the lack of capacity in pathology and radiology remained [26]. Therefore, would allocation of more resources have been an approach to overcome resource constrains for the investigatory units. Further, doing assessments on beforehand to try to understand each clinics capacity or assign specific clinics the main responsibility for patients under investigation for cancer, a form or reallocation method, could be have been valuable.

Another challenge was mislabelled and incomplete referrals, mainly from primary care. Such inappropriate referrals are reported to lead to increased patient volumes, reduce the efficiency of the service provided for patients with significant symptoms [28] and add to the crowding-out effect when implementing CPP, i.e. health professionals need to assign lower priority to other patient groups to prioritize cancer patients in the CPP [4, 8, 15, 24]. A way to overcome inappropriate referrals could be to develop guidance materials on how to do a proper labelling and complete referrals which could have been highlighted in educational discussions between physicians in investigatory units and primary care.

Another frustration expressed in the present study was that lead-times in the CPP manuals were perceived as unrealistic and/or medically unjustified. Participants stated that it was more important to them to do things correctly rather than quickly. This is interesting, as from the start of CPP implementation, it has been stated that it is difficult to apply the same standardized pathway when dealing with frail patients and those with comorbidities, thereby making a longer diagnostic interval occasionally necessary to ensure best treatment [4]. The manuals with recommended lead-times was developed nationally by diagnosis specific multi-professional expert teams [4], but perhaps the suggested lead-times needs to be assessed and revised on a regular basis and more information should be provided to the health professionals involved in CPP to be convinced about these lead-times.

Most of the seven themes that describe healthcare professionals’ experiences of the implementation of CPPs in investigatory units in the present study fit into the major domains of the CFIR [12]. For example, Damschroder et al. (2009) suggest that the characteristics of individuals involved in the implementation is one major domain that may affect the implementation of an intervention. They claim that changes in individual behaviour lead to organizational changes [12]. This aligns with the results of the present study, where the increased awareness of the end-users, namely the patients, among the health professionals in the investigatory units led to them changing their behaviour so that they collaborated more effectively, resulting in a smother and faster CPP. Furthermore, another major CFIR domain that is important for the success of CPP implementation is the inner setting, which includes features of the implementing organization. In the present study, the inner setting involved positive aspects such as pre-existing well-functioning work processes and support functions, but also negative aspects such as lack of staff and clinical equipment. However, lack of staff, clinical equipment, unjustified time-slots and incorrect referrals are also a part of another major dimension of the CFIR, i.e. the outer setting, which includes features of the external context, which the unit is unable to control. Another major domain in the CFIR is intervention characteristics. In the present study, the features of CPP were quite well defined and the participants had been involved in the process of developing the definitions of CPP, which probably contributed to the fact that most healthcare professionals working in the investigatory units experienced benefits with/as a result of the implementation of CPP.

### Strengths and limitations

A strength of our study is that it is one of the few qualitative studies investigating the CPP implementation process with a focus on the investigatory units, which are essential for the CPP process. That several researchers conducted the interviews and the analysis enabled more transparency and reduced the risk of personal biases [29]. The use of quotations in the results strengthens the validity [30]. A limitation is that the sample is quite small and predominantly drawn from just one of the 21 Regions in Sweden, which limits the generalizability to other settings in other regions or countries. While using a snowball recruitment strategy is convenient, it may have influenced the results, in that participants may have chosen a colleague who was known to be either positive or negative about the experience. However, the result indicates that both perspectives were raised and that data saturation was achieved and discussed. If the domains described in the CFIR would have been used to develop the interview guide and to deductively analyse data other themes might have emerged, however using a thematic data analysis allowed the researchers to seek patterns of data regardless of the CFIR [21].

## Conclusions

Health care professionals in investigatory units experienced that implementing CPP contributed positively, especially to better collaboration and an increased focus on the patient. However, lack of staff and equipment, as well as sometimes, unjustified time frames were frustrating.

## Supplementary Information

**Additional file 1:**

## Data Availability

Not applicable. The data will not be shared, as ethical approval for the study requires that the transcribed interviews are kept in locked files, only accessible to the researchers.

## Abbreviations

CPP: Cancer patient pathway.
