# Supplementary material for: Exploring experiences of implementing standardized cancer patient pathways within investigatory units – a qualitative study
Source: BMC Health Serv Res. 2021 Sep 8;21:933. doi: 10.1186/s12913-021-06915-1 (PMC8425083; doi:10.1186/s12913-021-06915-1)
Supplement: Supplementary file 1 — Additional file 1: [file 12913_2021_6915_MOESM1_ESM.docx]

**Interview guide in the study exploring experiences of implementing** **standardized cancer patient pathways (CCPs) within investigatory units.**

The 2017 version of the interview guide

General questions

- Can you shortly describe yourself and your main tasks at your workplace?
- In general, which are the strengths and challenges at your workplace?

Individual experiences of CCP

- Generally, what are your reflection about CCPs up to now?
- What does CCPs means for you?
- What is different now compare to before CCPs was implemented?
- What is the largest difference between working with CCPs compared to the usual way you are working?
- How secure to you feel when it comes to work according to CCPs guidelines?
- What do you think is the greatest success factor for CCPs?

Implementing CCPs at the workplace

- How is CCPs implemented in your current way of working?
- Did you feel involved when CCPs were implemented?
- Have you been supported in the implementation of CCPs?
- Which resources is needed to implement CCPS?
- How do you perceive your colleagues and managements attitude to CCPs?
- Have the implementation of CCPs influenced your workplace?
- What is working fine with CCPs and what is the most important factor to get CCPs functioning at your workplace?
- What challenges do you experience with CCPs?
- How do you perceive the cooperation between your workplace and others?

The CCPs influence on patients

- How do you think CCPs is perceived by patients?
- Do patients know anything about CCPs?
- Are there any pros and cons for patients?

General points of views on CCPs

- What challenges to you see with CCPs?
- What are the short- and long-term consequences of CCP?
- Do you think it is important to implement methods as CCP in health care?

Other

- Anything else you want to add?

The 2018 version of the interview guide

General questions

- Can you shortly describe a little bit about yourself and your main tasks at work?

Individual experiences of CCP

- How do you experience working with CCPs today?
- What is the largest difference to work with CCPs compared to the usual way you are working?*

CCPs at your workplace today

- What is working fine with CCPs and what is the most important factor to get CCPs functioning at your workplace?*
- What challenges to you experience with CCPs? *
- What is the largest strength with CCPs?
- How do you perceive the cooperation between your workplace and others?*
- What is your view of the managers roll when it comes to making CCPs work at your workplace?

The CCPs influence on patients

- Do you experience that patients are informed that they are included in an CCP? If not, how do you work with informing patients?

General points of views on CCPs

- What are the short- and long-term consequences of CCPs?*
- Do you think there is a problem with a crowding-out effect when implementing CCPs?

Other

- Anything else you want to add?*

* The question is also used both in 2017 and 2018 interview guide.
